# Apraxia of speech and cerebellar mutism syndrome: a case report

**DOI:** 10.1186/s40673-016-0059-x

**Published:** 2017-01-06

**Authors:** E. De Witte, I. Wilssens, D. De Surgeloose, G. Dua, M. Moens, J. Verhoeven, M. Manto, P. Mariën

**Affiliations:** 1 Clinical and Experimental Neurolinguistics, CLIN, Vrije Universiteit Brussel, Pleinlaan 2, B-1050 Brussels, Belgium; 2 Department of Speech and Language Therapy, ZNA Middelheim, Lindendreef 1, B-2020 Antwerp, Belgium; 3 Department of Radiology, ZNA Middelheim, Lindendreef 1, B-2020 Antwerp, Belgium; 4 Department of Neurosurgery, ZNA Middelheim, Lindendreef 1, B-2020 Antwerp, Belgium; 5 Department of Neurosurgery and Center for Neuroscience, Universitair Ziekenhuis Brussel, Laarbeeklaan 101, B-1090 Brussels, Belgium; 6 Department of Language and Communication Science, City University London, Northampton Square, London, EC1V 0HB UK; 7 Unité d’Etude du Mouvement, FNRS-ULB, Bruxelles, Belgium; 8 Department of Neurology & Memory Clinic, ZNA Middelheim Hospital, Lindendreef 1, B-2020 Antwerp, Belgium

**Keywords:** Cerebellar mutism syndrome, Posterior fossa, Apraxia of speech, Ataxic dysarthria, Cerebellum, Medulloblastoma, Case report

## Abstract

**Background:**

Cerebellar mutism syndrome (CMS) or posterior fossa syndrome (PFS) consists of a constellation of neuropsychiatric, neuropsychological and neurogenic speech and language deficits. It is most commonly observed in children after posterior fossa tumor surgery. The most prominent feature of CMS is mutism, which generally starts after a few days after the operation, has a limited duration and is typically followed by motor speech deficits. However, the core speech disorder subserving CMS is still unclear.

**Case presentation:**

This study investigates the speech and language symptoms following posterior fossa medulloblastoma surgery in a 12-year-old right-handed boy. An extensive battery of formal speech (DIAS = Diagnostic Instrument Apraxia of Speech) and language tests were administered during a follow-up of 6 weeks after surgery. Although the neurological and neuropsychological (affective, cognitive) symptoms of this patient are consistent with Schmahmann’s syndrome, the speech and language symptoms were markedly different from what is typically described in the literature. In-depth analyses of speech production revealed features consistent with a diagnosis of apraxia of speech (AoS) while ataxic dysarthria was completely absent. In addition, language assessments showed genuine aphasic deficits as reflected by distorted language production and perception, wordfinding difficulties, grammatical disturbances and verbal fluency deficits.

**Conclusion:**

To the best of our knowledge this case might be the first example that clearly demonstrates that a higher level motor planning disorder (apraxia) may be the origin of disrupted speech in CMS. In addition, identification of non-motor linguistic disturbances during follow-up add to the view that the cerebellum not only plays a crucial role in the planning and execution of speech but also in linguistic processing. Whether the cerebellum has a direct or indirect role in motor speech planning needs to be further investigated.

## Background

Cerebellar mutism syndrome (CMS) or posterior fossa syndrome (PFS) is characterized by a transient period of cerebellar mutism combined with a range of neurological (motor) disturbances, neuropsychological (cognitive, affective/behavioral) abnormalities and neurogenic speech/language deficits. It is most commonly observed in children and has only been occasionally reported in adults [1–4]. CMS is typically associated with posterior fossa tumor surgery [2], but traumatic brain injury [5], stroke [2, 6] and infections [7, 8] may also cause this syndrome. In a review of 257 children who developed CMS after surgery [9] 62.7% of the cases had a medulloblastoma, 24.9% an astrocytoma, 11.2% an ependymoma, 0.4% a meningioma (ME), 0.4% a germinoma, and 0.4% germ cells. The symptoms of CMS are linked to damage to various parts of the cerebellum and the cerebello-cerebral pathways passing through the brainstem [10]. Immediately after surgery, most patients who later develop CM present ataxia and dysmetria. After a linguistically symptom free interval of some days (generally 1 and a half to 2 days) the patients become mute [9]. Onset of CM is typically accompanied by additional neurological signs and affective/behavioral disorders. Neurological symptoms may consist of oculomotor and oral motor dysfunction, hypotonia, ataxia, paresis, dysmetria, incontinence, tremor and 6th and 7th cranial nerve palsies [11, 12]. Affective/behavioral disorders include irritability and whining (2/3) and apathy (1/2) and tend to resolve before remission of CM [13, 14]. After a period of CM (mean time: 47.6 days/43 days) [9, 15], speech slowly returns and is often characterized by distorted vowels, slowed speech, prolonged phonemes, monopitch/monoloudness, hypernasality, vocal tremor and dysarthria [13, 15–19]. Dysarthria which characterizes motor speech in most of the children after remission of mutism (98.8%) [15, 20] is typically defined as ataxic dysarthria [20]. Only a handful of studies reported that patients affected by CM had non-motor language problems such as a reduced verbal output with short phrases, wordfinding difficulties and disruption of grammar [14, 20, 21]. Neuropsychological studies revealed a range of executive dysfunctions, visuospatial problems and a general intellectual decline during longitudinal follow-up (even 1 year postsurgery) [20, 22]. The residual complex of cognitive, linguistic and affective symptoms in pediatric posterior fossa tumor patients are consistent with the Cerebellar Cognitive Affective Syndrome (CCAS) or Schmahmann’s syndrome [21, 23–25]. Schmahmann’s syndrome consists of a cluster of neuropsychological and affective impairments but do not per se include motor symptoms or CM [26]. However, it might be argued that CM represents the extreme end of adynamic speech on a fluency/dysfluency continuum, which forms part of the linguistic cluster of Schmahmann’s syndrome.

The pathophysiological mechanisms subserving CMS remain to be clarified. The immediate onset of ataxia and dysmetria are likely due to surgical impact on the cerebellum. Secondary processes initiated by the tumor resection seem to play a role in the delayed onset of symptoms [11, 27] and may involve edema of the cerebellum and the cerebellar peduncles, hypoperfusion and subsequent ischemia of the cerebellum, transient dysregulation of neurotransmitter release, crossed cerebello-cerebral diaschisis, and axonal injury. Diaschisis affecting supratentorial brain areas may indeed explain some cognitive symptoms of CMS. Mariën et al. [28] showed that lesions of the right cerebellar hemisphere may result in functional deactivation of supratentorial language areas in the left cerebral hemisphere, and introduced the concept of a ‘lateralized linguistic cerebellum’. Hypoperfusion in frontal regions due to cerebellar lesions may lead to grammatical and executive disorders [29]. SPECT (Single-Photon Emission Computed Tomography) studies have shown hypoperfusion in dominant or bilateral frontal regions during mutism and improvement in blood flow when speech returned [20, 28].

Following remission of CM the cluster of motor speech characteristics are often defined as ataxic dysarthria, since this type of dysarthria is typically found in a context of cerebellar pathology [29]. However, there is no clear-cut typology, which unambiguously classifies the motor speech symptoms as ataxic dysarthria. In an auditory-perceptual analysis of the speech characteristics of 24 children and adolescents, De Smet et al. [16] showed that typical ataxic speech characteristics such as imprecise consonants, excess and equal stress, and irregular articulatory breakdown were only present in a minority of the patients (fewer than 25%). Distorted vowels not associated with irregular articulatory breakdown and/or excess and equal stress were found in seven of the 13 (54%) patients, which confirms that this speech characteristic per se is not distinctive of ataxic dysarthria. As a result, the exact nature of motor speech pathology of CMS remains to be elucidated.

This study investigates the speech and language symptoms following posterior fossa medulloblastoma surgery in a 12-year-old right-handed boy. A detailed description of the motor speech characteristics may help to unravel the puzzling nature and pathophysiology of the CMS.

## Case presentation

### Case history

Following a 1-year period of frequent episodes of headaches, neck pain and progressive coordination and balancing problems, a 12-year-old right-handed boy (LD) was admitted to hospital. Medical history consisted of repeated falls with a brief loss of consciousness at the age of 2 years. A neurological and cardiological work-up including repeat EEG (electroencephalography) and ECG (electrocardiogram) revealed no abnormalities. Growth and developmental milestones were reported normal. He was born at term after normal gestation and labor and there had been no perinatal or postnatal problems. His scholastic achievements had always been above average levels and there was no familial history of developmental disorder or learning disability. Both his parents and two brothers (14 and 16 years old) were healthy.

**Fig. 1:**
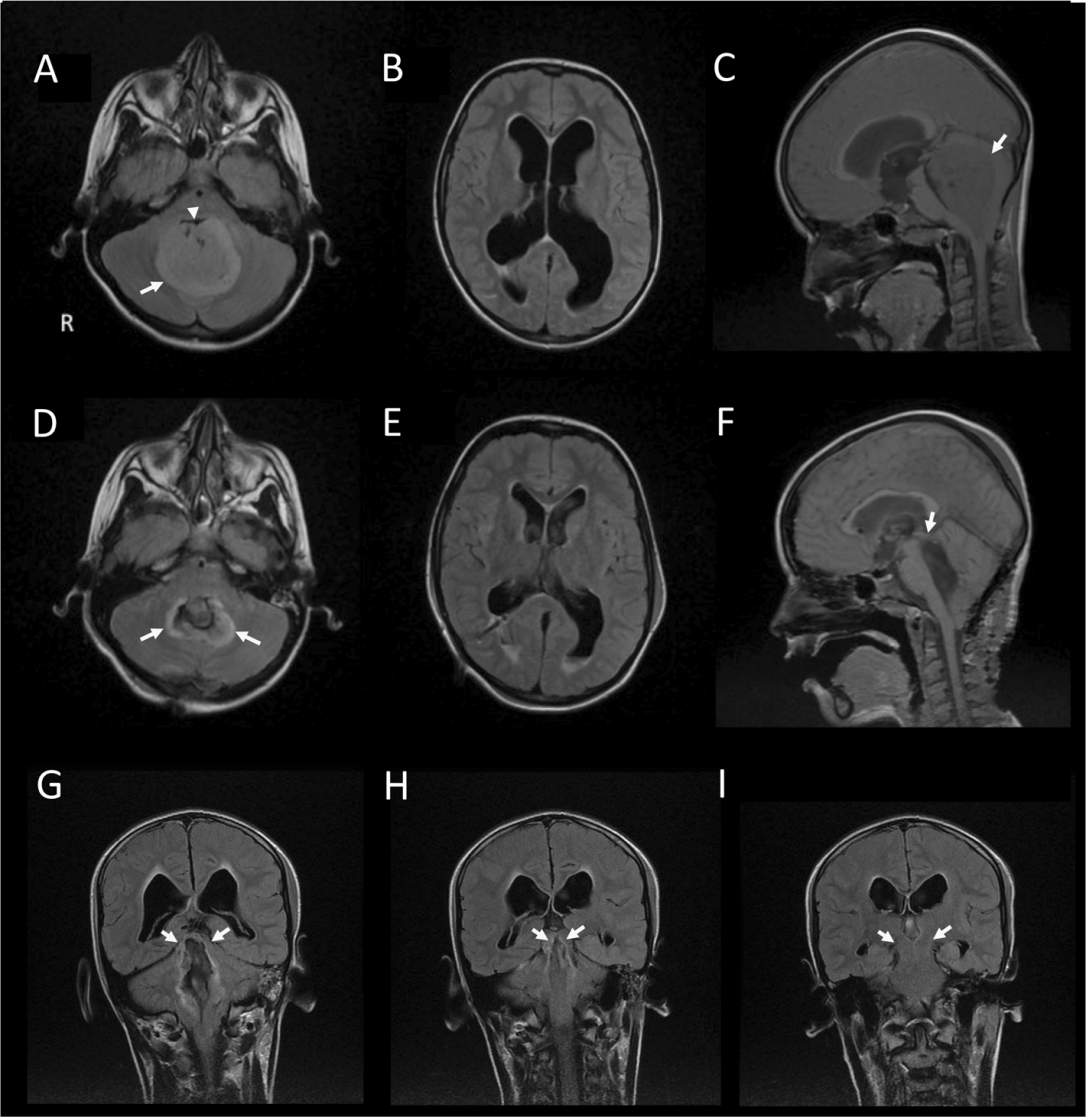
(**a-i**) Preoperative brain MRI (**a-c**). The *white arrow* (**a**; axial Flair sequence) points to the tumor invading the cerebellar parenchyma bilaterally. The lesion appears slightly hyperintense. The 4th ventricle is invaded (*white arrowhead*), causing a hydrocephalus (dilatation of the lateral ventricles in **b**). The tumor expands clearly in the vermis as shown on a sagittal image (**c**). Early postoperative axial FLAIR slice shows postoperative sequelae at the level of dentate nuclei bilaterally (*white arrows* in **d**). The hydrocephalus is resolved (**e**). The 4th ventricle is moderately dilated, including in the rostral direction. The superior medullary velum is visible (*white arrow* in **f**). The superior cerebellar peduncles are involved (*white arrows* in **g**), with a slight extension towards the corpora quadrigemina at the level of inferior colliculi (*white arrows* in **h**). Crus cerebri are spared bilaterally (*white arrows* in **i**). R: right side

The neurological examination on admission revealed mild hypotonia, dysmetria (finger-to-nose test) and balancing problems (Romberg test). No speech or language problems were observed but in-depth linguistic assessments were not performed. MRI (Magnetic Resonance Imaging) of the brain showed a tumoral mass lesion in the fourth ventricle extending to the foramen magnum, invading the vermis and cerebellar parenchyma bilaterally and exerting mass effect on the tentorium, pons and cerebellum (Fig. 1a-c). The third and lateral ventricles were enlarged due to secondary obstructive hydrocephalus. A ventricular drain was installed and the tumor was removed following a surgical incision from the inion to C7 and of the vermis of 3 centimetres. Subtotal tumor resection was achieved because of tumoral invasion in the brainstem. Anatomopathological examination disclosed a medulloblastoma (grade IV).

### Postoperative findings: Day one - day four

Early postoperative MRI of the brain revealed subtotal tumor resection with tumoral residue located posteriorly right to the resection cavity, cranial to the vermis. Remission of the hydrocephalus was observed. However, postoperative sequelae included tissue damage at the level of both caudate nuclei, moderate dilation of the 4th ventricle, involvement of the cerebellar peduncles with a slight extension towards the corpora quadrigemina at the level of the inferior colliculi (Fig. 1d-i). Reduction of ventricular enlargement was found as well. In the first 4 days after surgery, the patient adequately produced only a few words (e.g. ‘yes’, ‘mother’) and some short sentences (‘I have to pee’, ‘I know that’). Behavioral initiation was markedly reduced. Drip food was given as oral intake was impossible because of insufficient oral control and sialorrhea.

Clinical neurological examination showed asymmetric peripheral muscle weakness (more severe at the right side than at the left side), but the peripheral reflexes were symmetrically preserved and cranial nerves were unimpaired. Ataxia, hypotonia, dysmetria and akinesia were found. He developed a general state of apathy during the first days after surgery.

### Postoperative findings: Day five - week three

Due to leakage of cerebrospinal fluid at the insertion point of the drain and increased hydrocephalus, a new external drain was installed in combination with a ventriculostomy 2 weeks after surgery. The drain was removed one week later. On repeat CT 1 month after surgery (7/10) the ventricular volumes had diminished.

At day 5, he developed akinetic mutism. Auditory comprehension for daily conversation seemed intact since he succeeded to accurately answer yes/no questions in a non-verbal but slightly delayed way using his hands. He had marked difficulties moving his mouth, tongue and eyelids on request (voluntary movements) although automatic movements were carried out flawlessly (e.g. when swallowing or during spontaneous eye-blinking). He could not produce any sounds on command but when coughing or laughing, phonation was clearly audible. Marked presence of automatico-voluntary dissociations clearly indicated buccolabiolingual and eyelid apraxia. A bilateral paresis of the trochlear nerve (right > left) was present and a right paresis of the abducens nerve induced diplopia. After 1 week postsurgery, speech therapy was started to practice orofacial movements and to initiate speech. Under the guidance of the speech therapist peroral food was slowly built up successfully 2 weeks after surgery. Physiotherapy was started which resulted in increased movements of the arms and legs. Ataxia, hypotonia and akinesia were still present at 3 weeks postsurgery.

Aside from apathy, he displayed emotional instability, infantile behavior and he was extremely frustrated and unhappy not being able to speak. A psychiatric evaluation three weeks after surgery confirmed that frustration occurred secondary to mutism.

### Postoperative findings: week four - week six

Four weeks after surgery the boy gradually started to speak again. Spontaneous output was severely adynamic (few words/sentences) at week four but became more fluent in the following weeks. The patient spoke with a clear dental articulatory setting: all alveolar sounds (/t/, /d/, /n/ and /l/) were consistently pronounced with the tongue tip against or between the teeth. In addition, the patient had a falsetto voice quality with an average fundamental frequency of 490 Hz. The patient was able to lower his voice on command, but this sounded forced and unnatural. Speech was further characterized by the prolongation of speech sounds (e.g. ‘kammmm’ instead of ‘kam’ (comb)), the devoicing of consonants (e.g. ‘pank’ instead of ‘bank’ (bank), mostly in initial position), hypernasality and self-corrections. In addition, speech tempo was relatively slow (2.5 syllables per second). This is significantly slower than in a reference sample of adult male and female speech in Verhoeven et al. [30] in which articulation rate amounted to 4.23 syllables per second. The speaker’s speech rhythm was quantified by means of the pairwise variability index (PVI) proposed by Ling et al. [31]. This index is based on measurements of vowel duration (vocalic PVI) and the duration of the intervocalic intervals (intervocalic PVI). In this patient, the vocalic PVI amounted to 33.4: this is considerably lower than 65.5, which is the reference value for Dutch suggested in Grabe and Low [32]. However, PVI is between that of French (43.5) and that of Spanish (29.7). This indicates that the patient’s speech rhythm is more syllable-timed than the Dutch stress-timing and this confirms the overall auditory impression of staccato speech.

Aside from staccato speech, none of the typical signs of (ataxic) dysarthria such as distorted vowels, imprecise consonants, excess and equal stress, and irregular articulatory breakdown were observed. By marked contrast, typical characteristics of apraxia of speech (AoS) were found including articulatory groping and segmentation. Formal investigation of motor speech by means of the Diagnostic Instrument Apraxia of Speech (DIAS) [33] was performed at six weeks postsurgery. The DIAS consists of a set of eight tasks that measure the typical characteristics of AoS (Table 1). The tasks include: performance of buccofacial movements (on command, after imitation), repetition of consonants and vowels, repetition of sequential and alternating sequences (diadochokinesis) and repetition of words (non-compounds, compounds, with/without consonant clusters). If three or more characteristics (Ca) are present, AoS is diagnosed. In this patient deviant speech features included a much worser performance for alternating sequences than for sequential sequences (Ca3), articulatory groping (Ca4), initiation problems (Ca5), syllable segmentations (Ca6), cluster segmentations (Ca7), and an articulation complexity effect (Ca8). As six out of the eight typical features were met, AoS was diagnosed (Table 1). Although buccolabiolingual praxis had improved, apraxia was still formally diagnosed by means of the DIAS [33]; articulatory groping was observed and the performance of articulatory movements improved when imitating (see Table 1).

**Table 1:** Speech/language/cognitive assessments

TASKS	Raw score (Max)	Z-score/ Pc/ Cut-off score
SPEECH		
DIAS		
buccofacial apraxia	5	C > 2
C1: inconsistent production	2	C > 2
C2: worse performance for the Cn than for the V	2	C > 2
C3: worse performance for the AS than for SS	0.8	C < 0.72^a^
C4: articulatory groping	3	C > 0^a^
C5: initiation problems	4	C > 0.09^a^
C6: syllable segmentations	2	C > 0^a^
C7: cluster segmentations	2	C > 0^a^
C8: articulation complexity effect	0.92	C > 0.88^a^
LANGUAGE		
ScreeLing		
- phonological index	24 (24)	C <19
- semantic index	21 (24)	C < 19
- syntactic index	18 (24)	C < 19^a^
CELF		
- Receptive language index	26 (79)	Pc = 8.1^a^
- Expressive language index	25 (77)	Pc = 6.3^a^
- Language content index	18 (68)	Pc = 1.6^a^
- Language form index	18 (76)	Pc = 5.5^a^
BOSTON NAMING TEST-NL	38 (60)	Z = −2.22 ^a^
EXECUTIVE FUNCTIONS (also language)		
- Phonological fluency	4	Z = −2.90^a^
- Semantic fluency	8	Z = −3.10^a^
VERBAL WORKING MEMORY		
CELF		
- Language memory index	7 (60)	Pc = 0.4^a^

*Pc* Percentile, *DIAS* Diagnostic Instrument Apraxia of Speech, *C* characteristic, *Cn* consonants, *V* vowels, *AS* alternating sequences, *SS* sequential sequences *CELF* Clinical Evaluation of Language Fundamentals, *NL* Dutch version

^a^defective results

Additional linguistic and cognitive assessments were carried out at 6 weeks postsurgery on the basis of the ScreeLing [34], the Clinical Evaluation of Language Fundamentals (CELF) [35], the Boston Naming Test-NL (BNT) [36, 37] and verbal fluency tasks (phonological, semantic fluency) (Table 1). The ScreeLing revealed normal phonological (24/24, cut-off 19) and semantic functions (21/24 cut-off 19) while syntax (18/24, cut-off 19) was impaired. The CELF yielded defective results on all five indexes (receptive language index: percentile 8.1, expressive language index: percentile 6.3, language content index: percentile 1.6, language form index: percentile 5.5, language memory index: percentile: 0.4). Visual confrontation naming (BNT: 38/60, z = −2.22) and verbal fluency (phonological: z = −2.90, semantic: z = −3.10) were also impaired (see Table 1 for detailed results). In summary, linguistic test results indicated a general language production/perception impairment (CELF, indexes 1–4) with prominent wordfinding difficulties (BNT) and syntactic impairments (ScreeLing). In addition, he presented verbal working memory deficits (CELF, index 5) and verbal executive dysfunctions (verbal fluency).

Although the right hemiparesis improved, the boy was bounded to a wheelchair. Hypotonia, ataxia and diplopia persisted. Affective/behavioral symptoms diminished but overall frontal behavioral disinhibition with infantile features (continuously laughing, (falsetto voice), interdental articulatory setting) were still present 6 weeks after surgery. The infantile behavior decreased when the family was not present in the room.

### Postoperative findings: week seven - month six

A grade IV medulloblastoma was treated by means of a program of radiotherapy (boost of 30.6Gy in 17 extra fractions of 1.8 Gy) and a preventive program of craniospinal radiotherapy (23.4Gy in 13 fractions of 1.8Gy). During the radiotherapy concomitant chemotherapy was given (Vincristine 1.5 mg/mt 1×/week * six cycli). During the entire period of radio and chemotherapy patient suffered from frequent headaches, nausea and infections which made extensive cognitive assessment impossible.

He was admitted to a rehabilitation center in between the four cycli of chemotherapy where he entered a multidisciplinary therapy program (speech therapy, physiotherapy, ergotherapy). Clinical neurological investigations at 6 months postsurgery showed almost symmetrical strength with remission of the right hemiparesis. The patient started walking again but ataxia remained. Speech and language improved but slowed speech, articulatory groping, segmentation and wordfinding difficulties were still observed. No formal language tests were performed. He spoke with a normal tonal voice but the falsetto voice reappeared occasionally. After 8 months planned neurocognitive examinations were cancelled because of regrowth of the tumor.

## Discussion

This patient with CMS not only shares a number of overt similarities but also some differences with the complex of symptoms that constitute CMS as generally described in the literature. As in most cases with CMS, this patient developed cerebellar mutism (CM) combined with neurological symptoms, neuropsychological disturbances and speech deficits following posterior fossa tumor surgery [1–4]. Postoperatively, a spectrum of neurological symptoms were found that consisted of: ataxia, dysmetria, hypotonia, akinesia, diplopia (trochlear and abducens paresis) and a bilateral paresis which affected the right body-side more prominently. The boy became mute 4 days after partial resection of a medulloblastoma in the posterior fossa. During these first 4 days after surgery, reduced verbal output and a general state of apathy were found. The onset of CM was accompanied by apraxic deficits (oral and eyelid apraxia) as well as affective and behavioral disturbances (emotional instability, infantile behavior, irritability). After 4 weeks of CM, speech slowly returned. Although behavioral and affective symptoms diminished, infantile behavior persisted. At 6 weeks postsurgery, formal assessment of speech (DIAS) was consistent with a diagnosis of AoS. Ataxic dysarthria was formally excluded since no vowel distortions, imprecise consonant production, excess and equal stress and irregular articulatory breakdown were found. The neurolinguistic work-up further showed distorted language production and perception, wordfinding difficulties, grammatical disturbances and verbal fluency deficits. After 6 months, speech and language as well as motor skills improved but did not return to preoperative level.

Although the neurological and neuropsychological (affective, cognitive) symptoms of this patient are typical for CMS [13–20, 22], speech and language symptoms were markedly different from what is typically described in the literature [20]. Non-motor language deficits are only rarely reported in the CMS literature. Indeed, after a careful review of 167 cases published between 1972 and 2006, De Smet et al. [15] identified 165 (98.8%) patients with postoperative motor speech disorders and only a handful of cases with non-motor language deficits [38]. The language deficits described in the study of Riva and Georgi [38] consisted of agrammatic, hypospontaneous language. However, language symptoms in the CMS population have not been systematically investigated by means of formal instruments and might therefore have been overlooked [28]. In this patient syntactic, wordfinding and verbal fluency deficits were identified as the most prominent language deficits. Only two studies similarly described language deficits following posterior fossa tumor surgery. In the study of De Smet et al. [20] 4 patients affected by CM suffered from adynamic speech production, impaired verbal fluency, word-finding difficulties and grammatical disturbances at 1.5–6 months postoperatively. Levisohn et al. [21] showed that 11 out of 19 posterior fossa tumor patients (regardless of the presence/absence of CM) had word-finding difficulties/severe language problems within 2 years from surgery. The linguistic symptoms seem to form part of the Cerebellar Cognitive Affective Syndrome (CCAS) or Schmahmann’s syndrome [21, 23–25] that refers to a constellation of neuropsychological (visuo-spatial, executive), affective and linguistic impairments that are caused by cerebellar lesions. PFS, CM, CCAS are related conditions that have been used interchangeable in studies, which makes it hard to compare the results of different studies.

In the posterior fossa literature motor speech symptoms following CM are typically defined as ataxic dysarthria in posterior fossa literature [13, 15–19]. Reviewing the data on 283 childhood cases to chart the mode of recovery of motor speech production after CMS, De Smet et al. [20] showed that 98.8% of the reviewed cases displayed motor speech impairments. However, perceptual speech analysis in 24 children and adolescents (of whom 12 developed CMS) disclosed that the most prominent speech deficits consisted of distorted vowels, slow rate, voice tremor, and monopitch and that typical ataxic speech characteristics such as imprecise consonants, excess and equal stress, and irregular articulatory breakdown were only present in half of the patients (54%) [16]. De Smet et al. [16] concluded that motor speech disturbances following cerebellar tumor surgery do not necessarily resemble ataxic dysarthria. Acoustic/phonetic speech analyses and DIAS assessment [33] in this patient revealed the following characteristics: high pitch (falsetto voice), dental articulatory setting, hypernasality, prolongation of phonemes, slowed/staccato speech, devoicing of consonants, frequent self-corrections, seeking behavior/articulatory groping, initiation problems, syllable segmentations, cluster segmentations, worse performance for alternating sequences than for sequential sequences and an articulation complexity effect. Although hypernasality, prolongation of phonemes and slowed staccato speech are symptoms that overlap between ataxic dysarthria and AoS, there is ample evidence to conclude that this patient’s deviant speech production is consistent with AoS in the absence of ataxic dysarthria [29, 39]. First, DIAS results clearly matched a typological diagnosis of AoS as six out of the eight typical AoS features were met (seeking behavior/articulatory groping, initiation problems, syllable segmentations, cluster segmentations, worse performance for alternating sequences than for sequential sequences and an articulation complexity effect). Second, patients with AoS typically have problems with irregular voicing or devoicing [40] and frequently correct themselves [41], which was also the case in this patient (devoicing of consonants, self-corrections). Third, the three most typical symptoms of ataxic speech (i.e. imprecise consonants, excess and equal stress and irregular articulatory breakdown) were not observed in this patient. The origin of the high pitch falsetto voice and dental articulatory setting are unclear. These symptoms are neither typical of AoS nor of ataxic dysarthria. Tumor surgery may cause vagal neuropathy and as such vocal fold paralysis [42] inducing a compensatory falsetto or paralytic falsetto [43, 44]. However, ENT (ear, nose, throat) investigations did not reveal vagal neuropathy or vocal fold paralysis. The falsetto voice and the dental articulatory setting might therefore reflect phenomena linked to the childish/infantile behavior of this patient.

As a result it is hypothesized that the CM in this patient was more likely induced by an underlying speech planning and organization deficit (apraxia) and not by a pure motor disorder (dysarthria). AoS and ataxic dysarthria share some semiological similarities (e.g. irregularity, slowness), but these may reflect more universal aspects (e.g. motor slowness) [29]. The role of the cerebellum in ataxic dysarthria is generally acknowledged [45], whereas a possible role of the cerebellum in AoS remains to be elucidated. According to Ziegler [29] cerebellar lesions are not implicated in the origin of AoS. The primary lesion sites that have been assigned to AoS are Brodmann area 44 of Broca’s area, the left inferior premotor and motor cortex, and the left anterior insular cortex [46–48]. However, the aforementioned clinical symptoms of this case’s speech clearly match with the concepts of AoS put forward by Ziegler [29]: inconsistent speech movement aberrations resulting in inconsistent sound distortions and phonemic errors, slowed speech, breakdown of speech rhythm due to groping false starts, inter-/intrasyllable pauses, scanned rhythm, frequent self-correction. Consequently, in this case AoS was formally diagnosed and related to the cerebellar lesion. In addition, functional neuroimaging and clinical studies have recently shown that the cerebellum is also involved in speech motor control, which is required for the planning and execution of speech [49–52]. Nevertheless the exact role of the cerebellum in motor planning versus motor execution of speech is still unclear [29, 53]. Whether the cerebellum has a direct or indirect role in the motor speech planning network might be further investigated using SPECT reflecting distant functional effects in anatomically connected cerebral regions primarily involved in motor speech planning such as Broca's area, the language dominant inferior premotor and motor cortex and the anterior insula [46–48].

This case might be the first example that clearly demonstrates AoS following posterior fossa tumor surgery. Therefore, the findings add to the view that the cerebellum plays a crucial role in the planning and execution of speech. Whether the cerebellum has a direct or indirect role in motor speech planning needs to be further investigated. Although residual speech symptoms following CMS are frequently defined as ataxic dysarthria, there is no consensus on the core speech characteristics that follow CM [9, 12, 16, 54, 55]. Therefore, this case presenting apraxic rather than dysarthric speech deficits provides novel evidence that might add to current insights in the core features of motor speech and language disturbances in CMS.

### Limitations and future directions

No in-depth speech and language assessments were performed in the preoperative phase and after the 6 week follow-up period. No preoperative assessments were carried out because the presence of obstructive hydrocephalus required immediate surgical intervention. Longer-term postoperative assessments were missing due to referral to another hospital, time-consuming treatment plans and regrowth of the tumor. No motor scales were formally conducted but a close clinical neurological follow-up was performed. The use of functional neuroimaging might enable to further unravel the direct or indirect role of the cerebellum in motor speech planning.

## Conclusion

CMS consists of a constellation of neuropsychiatric, neuropsychological and neurogenic speech and language deficits. It is most commonly observed in children after posterior fossa tumor surgery. The most prominent feature of CMS is mutism, which generally starts after a few days after the operation, has a limited duration and is typically followed by motor speech deficits. On the pathophysiolgical level the complex range of symptoms unambiguously reflects crucial involvement of the cerebellum and the cerebro-cerebellar pathways in disrupted cognitive and affective processes. However, the core speech characteristics of CMS and the exact role of the cerebellum in motor speech planning versus execution are still unclear. In this article, a detailed description is provided of the motor speech and language deficits following posterior fossa tumor surgery in a 12-year-old right-handed boy. In-depth analyses of motor speech production revealed features consistent with a diagnosis of AoS while ataxic dysarthria was absent. Future research is needed to confirm these findings and to explore the precise role of the cerebellum in motor speech planning processes.

## Abbreviations

AoA: Apraxia of speech
BNT: Boston naming test
C: Cervicular
Ca: Characteristics
CCAS: Cerebellar cognitive affective syndrome
CELF: Clinical evaluation of language fundamentals
CM: Cerebellar mutism
CMS: Cerebellar mutism syndrome
CT: Computed tomography
DIAS: Diagnostic
ECG: Electrocardiogram
EEG: Electroencephalography
ENT: Ear nose and throat
Gy: Gray
MRI: Magnetic resonance imaging
NL: Nederlandse versie (Dutch version)
PFS: Posterior fossa syndrome
PVI: Pairwise variability index
SPECT: Single-photon emission computed tomography
